# Shifting the paradigm in bipolar disorder: moving beyond conventional inflammatory markers to novel EBI3-containing heterodimeric cytokines

**DOI:** 10.3389/fpsyt.2026.1759397

**Published:** 2026-08-25

**Authors:** Junfang Wu, Meixia Li, Long Cheng, Jun Chai, Shanshan Zhai

**Affiliations:** Department of Psychiatry, Taiyuan Central Hospital, Peking University First Hospital Taiyuan Hospital, Taiyuan, Shanxi, China

**Keywords:** bipolar disorder, EBI3, il-27, IL-35, IL-39

## Abstract

**Background:**

Research shows immune dysregulation in bipolar disorder (BD), but clinical heterogeneity and variable treatment responses persist around inflammatory markers like IL-6, TNF-α, and CRP. Newly identified EBI3-containing cytokines (IL-27, IL-35, IL-39) can be proinflammatory or immunoregulatory/putatively neuroprotective, and their role in BD remains unexplored.

**Methods:**

Serum levels of EBI3-containing inflammatory cytokines (IL-27, IL-35, IL-39, and EBI3) and conventional inflammatory markers (CRP, TNF-α, IL-6, and IL-10) were measured in this case-control, observational study using a high-sensitivity enzyme-linked immunosorbent assay (ELISA) in a cohort of 60 patients with acute mania and 60 healthy controls.

**Results:**

Compared with controls, patients with BD showed significantly higher serum levels of CRP (FDR-p = 0.008), IL-6 (FDR-p = 0.004), IL-10 (FDR-p = 0.008), IL-27 (FDR-p = 0.008), and IL-35 (FDR-p = 0.0027). TNF-α initially reached significance (p = 0.041) but did not remain significant after FDR correction (FDR-p = 0.0547). IL-39 (FDR-p = 0.473) and EBI3 (FDR-p = 0.713) showed no difference between patients and controls. These results highlight a pattern of heightened inflammatory and regulatory cytokine activity in BD.

**Conclusions:**

This study suggests that IL-27 and IL-35 dysregulation characterizes the acute manic state of bipolar disorder and reflects a possible imbalance between pro- and anti-inflammatory responses. While these EBI3-containing cytokines may hold promise as state-related markers, their broader relevance across mood phases remains to be determined. Further longitudinal research is needed to evaluate state- versus trait-dependent immune alterations and their potential implications for personalized immunomodulation in BD.

## Introduction

Bipolar disorder (BD) is a chronic, relapsing psychiatric condition characterized by repeated episodes of mania/hypomania and depression, leading to high morbidity and mortality rates (1). With a lifetime prevalence of approximately 2.4%, BD affects an estimated 50 million individuals globally and ranks among the top 10 causes of disability worldwide according to the World Health Organization, with healthcare costs exceeding $200 billion annually (2). Despite considerable advances in psychopharmacology, only 30–40% of BD patients achieve remission with standard mood stabilizers, necessitating identification of novel treatment targets and improved understanding of disease mechanisms (3). The underlying pathophysiological mechanisms of BD remain incompletely understood, limiting the development of more targeted and effective interventions (4). Emerging evidence has progressively shifted our understanding of BD’s etiology beyond classical neurotransmitter hypotheses toward a more integrative neurobiological model incorporating immune-inflammatory dysregulation as a fundamental component of disease pathogenesis (1, 5, 6).

The immune system plays a role in many neurological and psychiatric disorders, especially mood disorders (7, 8). Consistent findings of immune system disruptions across all phases of BD are reported from convergent meta-analyses and studies using brain imaging or postmortem samples (9, 10). Beyond the periphery, neuroimaging and postmortem investigations have revealed increased microglial activation and enhanced expression of proinflammatory markers in the central nervous system of BD patients (1, 5), indicating that immune dysregulation extends from the peripheral circulation to the brain parenchyma (4). Notably, patients with BD exhibit higher levels of peripheral blood pro-inflammatory markers, such as IL-6, TNF-α, and CRP, particularly during the manic phase (11, 12).

Historically, research on inflammation in BD have emphasized systemic involvement, focusing on classical pro- and anti-inflammatory mediators such as IL-6, TNF-α, IL-10, and IL-4 (9, 10, 13). Although these important studies have significantly advanced our understanding of immunologic involvement in BD, the significant clinical heterogeneity and varied responses to treatment can be observed not only between inflammatory subgroups but also within them, suggesting that traditional anti-inflammatory treatments do not fully address the complex immune mechanisms underlying this disorder (3, 9). The IL-12 family particularly merits investigation as these cytokines orchestrate T-helper differentiation patterns that may distinguish inflammatory subtypes of BD amenable to divergent therapeutic strategies (10, 14).

The IL-12 cytokine family includes IL-12 (p35/p40), IL-23 (p19/p40), IL-27 (p28/EBI3), and IL-35 (p35/EBI3), which perform essential functions in directing T-helper cell development as well as regulating adaptive and innate immune responses (15). Two of them, IL-27 and IL-35, share the Epstein-Barr virus-induced gene 3 (EBI3) protein as a common chain, with distinct subunits, forming a subset of regulatory immunomodulatory cytokines (16). Unlike the largely pro-inflammatory members of the IL-12 family, both IL-27 and IL-35 exhibit potent immunosuppressive and neuroprotective properties (14, 17). This contrasts sharply with traditional IL-6 and TNF-α studies, which measure individual soluble cytokines; the heterodimeric architecture of EBI3-containing cytokines confers distinct receptor specificity and signaling outcomes (18), potentially explaining why broad anti-inflammatory approaches have yielded modest clinical benefits in BD.

Interleukin-27, a specific immune mediator, stands out for its ability to restrict neuropathology on one hand and induce protective or regulatory immunity on the other (17). Additionally, IL-27 inhibits the differentiation of proinflammatory Th17 and Th1 cells (14). Mechanistically, IL-27-mediated neuroprotection has been shown to occur by suppressing the expression of granulocyte-macrophage colony-stimulating factor (GM-CSF) in CD4+ T cells and upregulating programmed death-ligand 1 (PD-L1) not only on CNS-resident cells but also on CNS-infiltrating myeloid cells (14, 19). Furthermore, findings from several reports demonstrate that IL-35 shares a similar action with IL-27 in inhibiting pro-inflammatory T cell responses and promoting regulatory T cells (mitigation) through partially overlapping yet independent molecular signaling pathways (14).

The roles of IL-27 and IL-35 in neuroprotection highlight the importance of EBI3-containing cytokines in regulating neuroinflammatory events. Besides the well-characterized EBI3-containing heterodimers, interleukin-39 (IL-39), a newly discovered member of the IL-12 family, is gaining attention in immune research (20). IL-39 consists of IL-23p19 and EBI3 subunits, sharing the EBI3 component with both IL-27 and IL-35 but exhibiting different functional properties (20, 21). Unlike the immunoregulatory nature of IL-27 and IL-35, IL-39 shows a proinflammatory phenotype and is mainly secreted by activated B cells after stimulation with lipopolysaccharide (20, 21). In mouse models of autoimmune diseases, such as systemic lupus erythematosus (SLE), IL-39 plays a pathogenic role; adoptive transfer of IL-39-producing B cells worsens disease symptoms, while deficiency in IL-39 subunits reduces disease severity (22). Although the relevance of IL-39 in psychiatric disorders has not yet been explored, its proinflammatory profile and connection to activated B cell responses may suggest a potential link to immune dysregulation in BD.

The intricate EBI3-associated heterodimer cytokine network may be disrupted in BD. Since inflammation has been implicated in the pathophysiology of BD and EBI3-containing cytokines are emerging as a potential class of mediators of neuroinflammation, we designed this study to assess these novel mediators (CRP, TNF-α, IL-6, IL-10, IL-27, and EBI3 along with routine inflammatory indicators) in patients with BD.

## Methods

### Study design and participants

Within a year, this case-control study was conducted at Taiyuan Central Hospital. The Ethics Committee and Review Board of Taiyuan Central Hospital authorized this study (approval No.: 2023.04.02). There were 60 BD patients and 60 age- and sex-matched healthy controls in the research. The DSM-5 was used by board-certified psychiatrists to diagnose BD patients. Healthy control subjects were recruited from the local community and underwent a standardized screening interview conducted by a trained psychiatrist to verify the absence of personal or family history of psychiatric illness. They were also screened for current psychiatric symptoms, medication use, smoking, alcohol consumption, and medical conditions that could influence immune or inflammatory status. Only individuals meeting all health criteria and reporting no medication use were included. All individuals provided written informed permission, and the institutional ethics committee authorized the research.

### Inclusion and exclusion criteria

Eligible participants were adults (≥18 years) who met DSM-5 criteria for bipolar disorder, currently experiencing an acute manic episode. All patients had experienced their first manic episode within the previous nine months and had no prior psychiatric hospitalizations or history of other major psychiatric disorders. Diagnosis was confirmed through a structured clinical interview performed by an experienced psychiatrist.

To ensure homogeneity and minimize confounding inflammatory influences, a detailed screening questionnaire was administered covering past and current medical comorbidities (including metabolic syndrome, obesity, autoimmune and inflammatory diseases, recent infection, trauma, or vaccination), current medication use (any psychotropic, immunomodulatory, corticosteroid, antibiotic, or NSAID treatment), and lifestyle factors such as significant sleep deprivation, smoking and alcohol consumption. Individuals reporting any of these conditions or exposures at the time of blood collection were excluded.

### Clinical assessment

The severity of the manic symptoms in the BD patients was assessed using the Young Mania Rating Scale (YMRS). The validated 11-item YMRS assesses content, disruptive-aggressive behavior rating of psycho-motor agitation or retardation (appearance), sexual interest, sleep, irritability, elevated mood, increased motor activity/energy, and speech (rate and amount). More severe mania is indicated by higher scores. Only values between 0 and 60 may be present in the total YMRS. Remission is indicated by a score of ≤12, mild range by a score of 13–19, minimal mania by a score of 20–25, moderate mania by a score of 26 but <38, and severe mania by a score of ≥38.

### Blood sample collection and laboratory analysis

Venous blood was collected between 9:00 and 11:00 A.M., after an overnight fast, and within the first 24 hours of hospital admission, before starting or modifying any psychotropic medications that could affect inflammatory parameters. After the fixation sample was obtained, the serum was held at -80 °C until it set, and clots were allowed to develop at room temperature for 30 minutes before being centrifuged at 1,500 × g for 10 minutes at 4 °C.

The electrolytes, lipid profiles, liver and renal function tests, thyroid function tests using enzyme-linked immunosorbent assay (ELISA), and whole blood counts using an automated cell counter were all assessed with standard automated analyzers. Serum CRP levels were measured using high-sensitivity nephelometry. Serum levels of TNF-α, IL-6, and IL-10 (BioLegend), IL-27 and EBI3 (Mabtech), and IL-35 and IL-39 (ELK Biotechnology) were quantified using commercial sandwich ELISA kits. The detection ranges were TNF-α (7.8–500 pg/mL), IL-6 (7.8–500 pg/mL), IL-10 (3.9–250 pg/mL), IL-27 (15.6–4000 pg/mL), IL-35 (15.6–1000 pg/mL), IL-39 (7.8–500 pg/mL), and EBI3 (10–10000 pg/mL). In each cytokine-specific kit, at least one of the antibodies (capture or detection) is raised against a unique subunit or epitope of the intended target (for example, the p28 subunit for IL-27 or the IL-12p35 subunit for IL-35), as specified by the manufacturers, thereby minimizing cross-reactivity with other EBI3-containing heterodimers.

ELISA, a standard sandwich immunoassay, was used to quantify cytokine levels. Briefly, a mouse monoclonal capture antibody specific for the target cytokine was immobilized on a 96-well plate. Following the addition of standards and samples, the target cytokine bound to the plate-bound capture antibody. A biotinylated detection antibody recognizing a distinct epitope of the same cytokine was then added to form an antibody–antigen–antibody “sandwich”. Avidin–horseradish peroxidase was subsequently applied to bind biotin, and Tetramethylbenzidine (TMB) substrate was used to generate a colorimetric reaction proportional to cytokine concentration. Absorbance at 450 nm was measured using a microplate reader after addition of stop solution, and cytokine concentrations were calculated against kit-specific standard curves.

### Statistical analysis

The statistical analyses were performed using SPSS version 27 (IBM Corp., Armonk, NY, USA). Continuous variables were reported as means ± standard deviations (SD), and categorical data as frequencies and percentages. Data normality was evaluated using the Shapiro–Wilk test and visual inspection of Q–Q plots. Because most cytokine and inflammatory marker variables showed non-normal distributions, group comparisons were conducted using the Mann–Whitney U test.

To control for potential false discoveries due to multiple testing across eight biomarkers, p-values were adjusted using the Benjamini–Hochberg False Discovery Rate (FDR) procedure (m = 8). Both the FDR-adjusted p-values and effect sizes (rank-biserial correlation, r = Z / √N) were reported. Median differences and their 95% confidence intervals were estimated through the Hodges–Lehmann procedure. Associations among novel cytokines and inflammatory markers were evaluated using Spearman’s rank correlation coefficient.

A two-tailed FDR-adjusted p-value < 0.05 was considered statistically significant.

## Results

### Demographic and clinical characteristics

A total of 120 participants were enrolled in this study, comprising 60 patients with bipolar disorder and 60 healthy controls. The mean age was 39.9 ± 10.8 years in the BD group and 38.3 ± 9.79 years in the control group. Male participants represented 38.3% (n=23) of the BD cohort and 45% (n=27) of the control cohort. Body mass index (BMI) was 25.36 ± 6.14 kg/m² in BD patients and 24.65 ± 5.72 kg/m² in controls.

Comprehensive laboratory evaluation showed overall comparable hematological, hepatic, renal, thyroid, and electrolyte parameters between patients with BD and healthy controls (**Table 1**). Serum triglyceride levels were slightly higher in BD patients (125.9 ± 88.1 mg/dL) compared to controls (116.9 ± 60.1 mg/dL), while AST (23.9 ± 11.0 U/L vs. 28.3 ± 24.3 U/L) and ALT (19.9 ± 10.8 U/L vs. 26.4 ± 31.1 U/L) values were modestly lower, all remaining within the normal clinical range. Other parameters—including complete blood counts, renal function tests, serum electrolytes, thyroid hormones, and lipid fractions (total, LDL, and HDL cholesterol)—did not differ appreciably between groups (see **Table 1** for detailed statistics). These findings indicate that the observed cytokine alterations are not attributable to systemic metabolic or hepatic abnormalities, underlining their specificity to immune-inflammatory dysregulation in BD.

**Table 1:** Baseline demographic and laboratory characteristics of BD patients and healthy controls.

Variable	Case n=60	Control n=60	P-value
Age	39.9 ± 10.8	38.3 ± 9.79	0.415
Sex (Men %)	23 (38.3%)	27 (45%)	0.332
BMI	25.36 ± 6.14	24.65 ± 5.72	0.278
WBC	7.75 ± 2.105	7.46 ± 2.330	0.361
RBC	4.55 ± 0.495	4.39 ± 0.430	0.176
HB	12.47 ± 1.374	12.65 ± 1.373	0.515
PLT	253.70 ± 81.992	235.55 ± 80.736	0.295
AST	23.88 ± 11.015	28.30 ± 24.335	0.931
ALT	19.85 ± 10.825	26.42 ± 31.065	0.793
ALKP	201.03 ± 64.379	197.18 ± 71.890	0.477
TSH	2.46 ± 1.994	2.69 ± 2.856	0.868
FT4	8.68 ± 1.816	8.53 ± 1.579	0.673
FT3	1.15 ± 0.281	1.13 ± 0.264	0.839
BUN	14.92 ± 4.469	15.25 ± 4.936	0.856
Cr	0.97 ± 0.204	0.99 ± 0.209	0.583
CPK	341.52 ± 545.260	296.88 ± 507.136	0.906
LDH	361.30 ± 134.828	340.92 ± 122.431	0.388
FBS	113.45 ± 37.363	117.72 ± 54.683	0.646
Bill T	0.83 ± 0.363	0.92 ± 0.720	0.479
Bill D	0.23 ± 0.103	0.25 ± 0.135	0.392
Na	140.93 ± 2.797	140.12 ± 3.765	0.442
K	3.95 ± 0.598	4.07 ± 0.402	0.410
P	3.85 ± 0.950	3.92 ± 0.958	0.543
Ca	9.31 ± 0.774	9.44 ± 0.830	0.145
Mg	2.04 ± 0.228	2.11 ± 0.246	0.148
ESR	17.77 ± 15.80	17.27 ± 16.41	0.873
TG	125.88 ± 88.089	116.85 ± 60.107	0.971
Total Cholesterol	172.87 ± 43.242	171.75 ± 49.949	0.671
LDL	96.52 ± 21.005	96.45 ± 25.379	0.769
HDL	42.23 ± 9.219	42.95 ± 10.248	0.676
Lym	29.03 ± 9.332	30.30 ± 10.393	0.493
Neutrophil	64.85 ± 11.202	64.42 ± 11.500	0.638

BMI, Body Mass Index; WBC, White Blood Cell count; RBC, Red Blood Cell count; HB, Hemoglobin; PLT, Platelet count; AST, Aspartate Aminotransferase; ALT, Alanine Aminotransferase; ALKP, Alkaline Phosphatase; TSH, Thyroid-Stimulating Hormone; FT4, Free Thyroxine; FT3, Free Triiodothyronine; BUN, Blood Urea Nitrogen; Cr, Creatinine; CPK, Creatine Phosphokinase; LDH, Lactate Dehydrogenase; FBS, Fasting Blood Sugar; Bill T, Total Bilirubin; Bill D, Direct Bilirubin; Na, Sodium; K, Potassium; P, Phosphorus; Ca, Calcium; Mg, Magnesium; ESR, Erythrocyte Sedimentation Rate; TG, Triglyceride; LDL, Low-Density Lipoprotein; HDL, High-Density Lipoprotein; Lym, Lymphocyte count.

### Comparison of inflammatory markers and novel cytokines between groups

**Figure 1:**
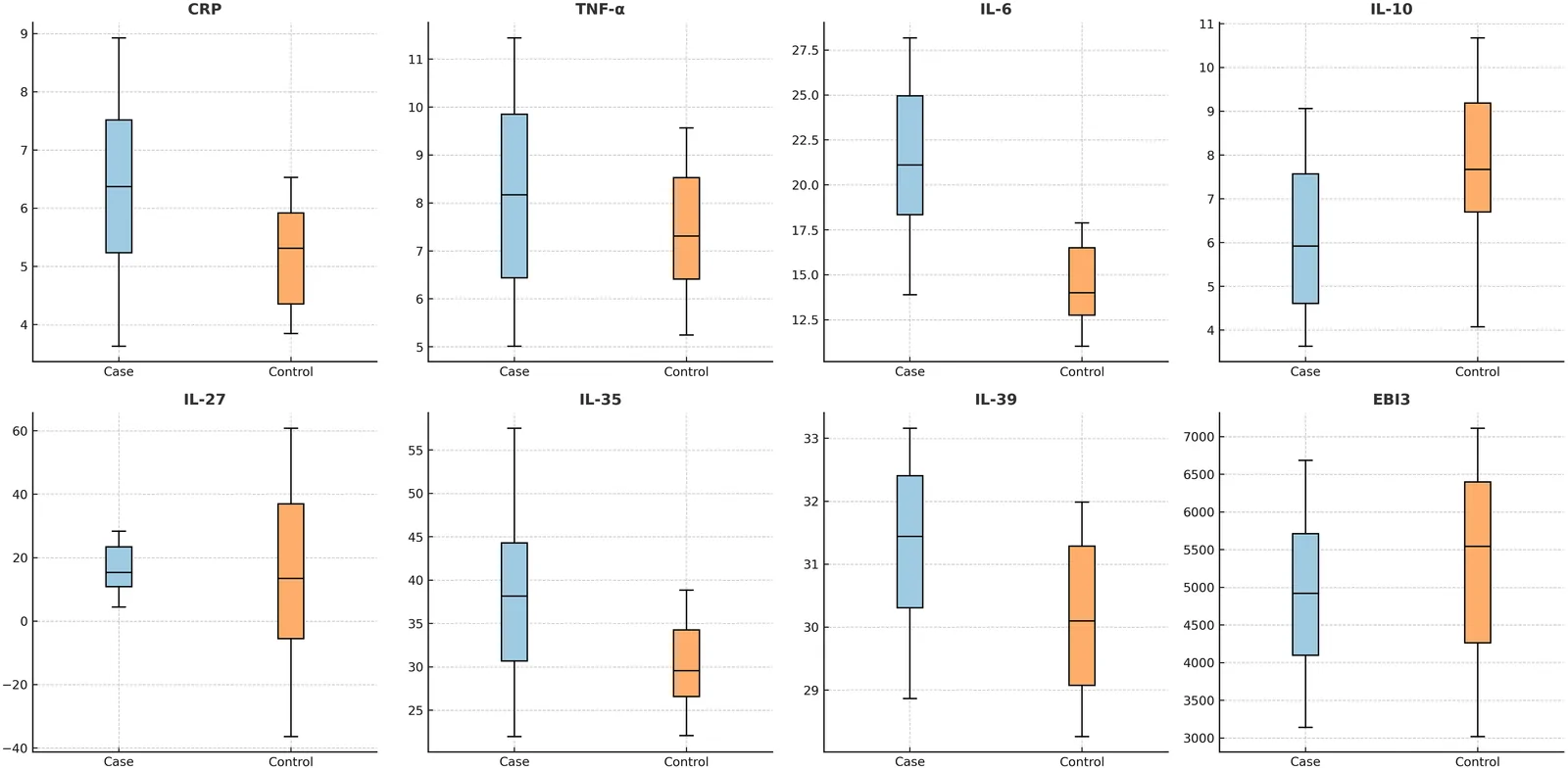
Comparison of serum inflammatory and cytokine levels in patients and controls. CRP, C-Reactive Protein; TNF, Tumor Necrosis Factor; IL-6, Interleukin-6; IL-10, Interleukin-10; EBI3, Epstein-Barr Virus-Induced Gene 3; IL-27, Interleukin-27; IL-35, Interleukin-35; IL-39, Interleukin-39.

Based on the Mann–Whitney U test followed by False Discovery Rate (FDR) correction, five out of eight examined biomarkers (CRP (0.008), IL-6 (0.004), IL-10 (0.008), IL-27 (0.008), and IL-35 (0.002)) remained significantly different between BD patients and controls. These cytokines demonstrated elevated or reduced median levels consistent with disease activity. TNF-α lost significance after FDR adjustment (0.054), while IL-39 (0.473) and EBI3 (0.713) levels showed no notable difference between the groups. Detailed statistics, including raw and FDR-adjusted p-values, effect sizes, and Hodges–Lehmann median differences with 95% confidence intervals, are presented in **Table 2**; **Figure 1**.

**Table 2:** Serum inflammatory and cytokine levels in patients and controls.

Variable Median (IQR)	Case n=60	Control n=60	P-value	FDR-adjusted p-value	r	HL (95%CI)
CRP *mg/L*	6.50 (5.75)	4.00 (2.88)	*< 0.001*	*≈ 0.008*	0.36	2.2 (1, 3.8)
TNF-a *pg/mL*	8.30 (6.775)	7.06 (4.335)	*0.041*	*0.0547*	0.18	1.5 (0, 3)
IL-6 *pg/mL*	21.20 (10.33)	6.50 (7.35)	*< 0.001*	*≈ 0.004*	0.57	13 (9.9,15.6)
IL-10 *pg/mL*	6.35 (5.50)	7.90 (7.70)	*0.005*	*0.008*	0.25	-2.5 (-4.1, -0.8)
IL-27 *pg/mL*	16.43 (15.5)	12.35 (6.98)	*0.004*	*0.008*	0.26	3.3 (0.8, 6)
IL-35 *pg/mL*	40.21 (36.575)	23.85 (17.08)	*< 0.001*	*≈ 0.0027*	0.45	14.95 (8.5, 22.2)
IL-39 *pg/mL*	31.01 (4.32)	30.13 (3.89)	*0.414*	*0.473*	0.07	1 (-1, 3)
EBI3 *pg/mL*	4882.50 (3841.50)	5264.47 (4418.75)	*0.713*	*0.713*	0.033	-146.5 (-1142, 800)

CRP, C-Reactive Protein; TNF, Tumor Necrosis Factor; IL-6, Interleukin-6; IL-10, Interleukin-10; EBI3, Epstein-Barr Virus-Induced Gene 3; IL-27, Interleukin-27; IL-35, Interleukin-35; IL-39, Interleukin-39; FDR– False Discovery Rate; HL (95% CI), Hodges–Lehmann with 95% Confidence Interval.

### YMRS-based grouping

11 patients in the present study had moderate (23–34) YMRS scores, while others exhibited severe mania (>37). Because no significant difference was observed between the groups in terms of inflammatory and cytokine profiles, severity of disease, which was also classified to YMRS scores, were not used as a covariate in the following analysis therefore, all patients were finally analyzed without distinction.

### Correlation analysis of inflammatory markers and novel mediators

**Figure 2:**
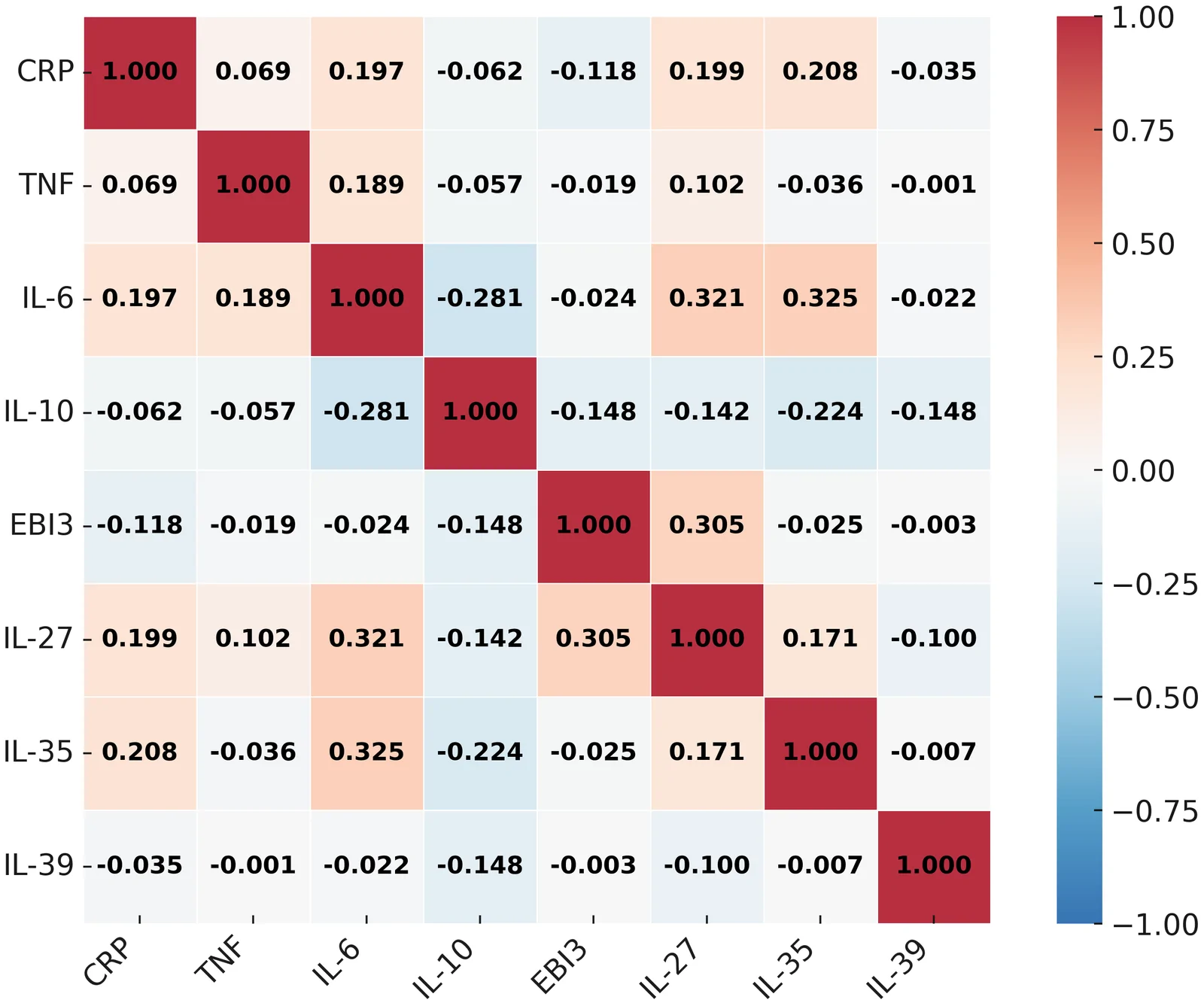
Spearman’s correlation matrix of serum inflammatory mediators in patients with bipolar disorder. Abbreviations: CRP – C-Reactive Protein, TNF – Tumor Necrosis Factor, IL-6 – Interleukin-6, IL-10 – Interleukin-10, EBI3 – Epstein-Barr Virus-Induced Gene 3, IL-27 – Interleukin-27, IL-35 – Interleukin-35, IL-39 – Interleukin-39.

Spearman’s correlation was used to analyze relationships between novel cytokines and inflammatory markers. CRP showed small but statistically significant positive correlations with IL-6 (r = 0.197, p = 0.031), IL-27 (r = 0.199, p = 0.030), and IL-35 (r = 0.208, p = 0.023). There was a small positive association between TNF-α and IL-6 (r = 0.189, p = 0.039). IL-6 also correlated significantly with other cytokines: a negative correlation with IL-10 (r = −0.281, p = 0.002) and small-to-moderate positive correlations with IL-27 (r = 0.321, p < 0.001) and IL-35 (r = 0.325, p < 0.001). IL-10 showed a weak inverse association with IL-35 (r = −0.224, p = 0.014). EBI3 was positively associated with IL-27 (r = 0.305, p < 0.001), whereas its expression had no evident correlation with IL-35 (r = −0.025, p = 0.785). The Spearman rank correlation between IL-27 and IL-35 was not statistically significant (r = 0.171, p = 0.062), and no association of IL-39 with other inflammatory mediators was found (**Table 3**; **Figure 2**).

**Table 3:** Spearman’s correlations among inflammatory and cytokines in BD patients.

		CRP	TNF	IL6	IL10	EBI3	IL27	IL35	IL39
CRP	Correlation Coefficient	1.000	0.069	0.197	-0.062	-0.118	0.199	0.208	-0.035
	P-value	.	0.452	0.031	0.498	0.200	0.030	0.023	0.700
TNF	Correlation Coefficient	0.069	1.000	0.189	-0.057	-0.019	0.102	-0.036	-0.001
	P-value	0.452	.	0.039	0.539	0.840	0.270	0.694	0.990
IL6	Correlation Coefficient	0.197	0.189	1.000	-0.281	-0.024	0.321	0.325	-0.022
	P-value	0.031	0.039	.	0.002	0.792	<0.001	<0.001	0.815
IL10	Correlation Coefficient	-0.062	-0.057	-0.281	1.000	-0.148	-0.142	-0.224	-0.148
	P-value	0.498	0.539	0.002	.	0.106	0.121	0.014	0.108
EBI3	Correlation Coefficient	-0.118	-0.019	-0.024	-0.148	1.000	0.305	-0.025	-0.003
	P-value	0.200	0.840	0.792	0.106	.	<0.001	0.785	0.971
IL27	Correlation Coefficient	0.199	0.102	0.321	-0.142	0.305	1.000	0.171	-0.100
	P-value	0.030	0.270	<0.001	0.121	<0.001	.	0.062	0.276
IL35	Correlation Coefficient	0.208	-0.036	0.325	-0.224	-0.025	.171	1.000	-0.007
	P-value	0.023	0.694	<0.001	0.014	0.785	0.062	.	0.939
IL39	Correlation Coefficient	-0.035	-0.001	-0.022	-0.148	-0.003	-0.100	-0.007	1.000
	P-value	0.700	0.990	.815	0.108	0.971	0.276	0.939	.

CRP, C-Reactive Protein; TNF, Tumor Necrosis Factor; IL-6, Interleukin-6; IL-10, Interleukin-10; EBI3, Epstein-Barr Virus-Induced Gene 3; IL-27, Interleukin-27; IL-35, Interleukin-35; IL-39, Interleukin-39.

Exploratory correlation analyses suggested possible interrelationships between some inflammatory markers and laboratory variables: TNF-α with HDL (r = −0.232, p = 0.011); IL-10 with RBC (r = 0.183, p = 0.045), HB (r = 0.256, p = 0.005), and BUN (r = −0.208, p = 0.023); and IL-39 with TSH (r = −0.188, p = 0.042) and phosphorus (r = 0.222, p = 0.015). However, given the small effect sizes, limited sample size, and exploratory nature of these analyses, these associations should be interpreted with caution, and no firm conclusions can be drawn. Larger cohort studies are warranted to clarify these relationships.

## Discussion

This is the first systematic investigation of EBI3-containing heterodimeric cytokines in a case-control bipolar disorder sample. Our findings reveal that IL-27 and IL-35, but not their shared EBI3 subunit, distinguish acute manic BD patients from healthy controls, suggesting that heterodimer assembly—rather than component subunit availability—may represent a regulatory checkpoint involved in BD immunopathogenesis. These results indicated a need for further specific investigation and clinical observation to assess the potential relevance of these immune mechanisms in the pathogenesis and treatment of BD.

CRP values were significantly higher in BD as compared to healthy controls, and this observation is consistent with that reported by a large meta-analysis conducted by Fernandes et al. (12) involving 1,618 participants from at least 11 studies and demonstrated significantly high levels of CRP in patients with BD (12). Critically, the current meta-analysis revealed that CRP levels are highest during mania, remaining moderately elevated in remission but failing to differentiate depression from control significantly (35, 36). Accordingly, the current sample comprising only acutely manic patients showed CRP levels within the higher range described in the literature for manic BD. ​Recent work by Ebrahim et al. (35) demonstrates that CRP is a consistent biomarker of systemic low-grade inflammation, and its elevation has been consistently associated with the acute manic phase and severe mood dysregulation (35). Consistently elevated CRP levels, seen even during stable BD states, indicate both state-dependent (acute) and trait-like immune dysregulation over the course of the illness BD (36, 37). Millett et al. (37) have reported robust associations of CRP levels with cognitive impairments in euthymic BD patients, indicating that CRP might be a biomarker for neurobiological impairment (37). These findings support the possibility that CRP may serve as an accessible inflammation marker in BD, although further verification in longitudinal datasets is required. However, the lack of longitudinal within-subject CRP trajectories in our dataset precludes assessment of whether CRP changes predict mood episode onset or resolution, limiting conclusions regarding its prognostic utility.

Our findings showed significantly higher levels of IL-6 in BD patients compared to controls, along with a less significant increase in TNF-α and IL-10. These results are strikingly consistent with the substantial Solmi et al. (11) study that showed increased IL-6 levels are a trait marker of BD, and it is more severe during an acute manic episode or depression (5). In another respect, in Brietzke et al. ‘s study (23), serum IL-6 levels were found to be increased in manic, depressive, and euthymic BD patients, but they were significantly higher during manic and hypomanic episodes (24, 25). Notably, Lu et al. (26) found that serum IL-6 level was significantly elevated in BD when compared with MDD during a similar depressive episode, suggesting the potential use of IL-6 for differential diagnosis (26). These findings raise the possibility that IL-6 elevation in our acute manic cohort may exceed that typically reported in major depressive disorder (MDD) at comparable disease severity, could potentially assist differential diagnosis between BD and MDD, pending confirmation by prospective comparative studies. However, prospective studies comparing IL-6 trajectories across mood phases in BD versus MDD are required to validate this hypothesis.

The small but consistent increase of TNF-α in our results aligns with several reports. Luo et al. (27) showed that not only the serum levels of TNF-α in manic, depressive, or mixed state BD patients were significantly higher than those in the normal control group, but they also mainly increased during acute mood episodes (27). Similarly, a comparative study between unipolar mania and bipolar disorder subjects, showed elevated levels of TNF-α in both groups, with greater increases during acute manic states compared to control conditions (18). Furthermore, the anti-inflammatory cytokine IL-10 was also significantly elevated in our results, a finding that is noteworthy considering previous literature by Modabbernia et al. (28), that observed increased levels of both pro- and anti-inflammatory signals, which continues to suggest a rather immunological imbalance rather than simple pro-inflammatory dominance (28). This indicates the shedding of immune factors to regulate systemic inflammation and may reflect a compensatory immunoregulatory response aimed at neutralizing systemic inflammation. Correlation analyses revealed small-to-moderate positive associations between IL-6 and both IL-27 and IL-35, suggesting a modest degree of coordinated variation in pro-inflammatory and immunoregulatory/putatively neuroprotective cytokine pathways. The negative correlation between IL-6 and IL-10 further supports the interpretation of a dysregulated but only modestly coupled immune balance in BD.

Surprisingly, among all EBI3 heterodimeric cytokines, IL-27 and IL-35 were significantly elevated, while IL-39 did not reach statistical significance. This finding adds new insights to BD immunology research, as IL-27 has been poorly described in BD samples. The pleiotropic cytokine IL-27 has potent anti-inflammatory and immunoregulatory/putatively neuroprotective properties by inhibiting the differentiation of pathogenic Th17 cells while promoting the development of IL-10-producing regulatory T cell responses (29, 30). Casella et al. (31) showed that IL-27 treatment modulates GM-CSF production by CD4+ T cells and inhibits neuroinflammatory disease in EAE (31). Similarly, Choi et al. (19) demonstrated that IL-27–producing B-1a cells mediate the inhibition of neurological inflammation and spontaneous autoimmune neurologic disease through different immunoregulatory mechanisms (19). Beyond its known anti-inflammatory and neuroprotective roles, Elevated IL-27 in BD is unlikely to directly reflect tissue-damaging or neuroinflammatory processes. It is possible that IL-27 modulates neuroendocrine axes or hormonal balance, as suggested by previous studies (32–34). However, paradoxically, the upregulation of IL-27 in our BD patients with immunoregulatory effects suggests that dysfunction of strong neuroprotective factors, rather than merely their simple deficiency, may be involved in the pathogenesis of BD. This observation can be compared with other neurodegenerative diseases, in which a similar compensatory upregulation of regulatory cytokines fails to suppress pathogenic inflammation adequately (17, 19). Correlation analyses revealed small-to-moderate positive associations between IL-6 and both IL-27 and IL-35, suggesting a modest degree of coordinated variation in pro-inflammatory and immunoregulatory/putatively neuroprotective cytokine pathways. The negative correlation between IL-6 and IL-10 further supports the interpretation of a dysregulated but only modestly coupled immune balance in BD. Prospective studies with serial assessments, both during acute and euthymic phases, are needed to explore the potential of IL-27 as a dynamic biomarker for determining illness state.

IL-35 levels were higher in BD patients than in healthy controls, with the most significant difference observed across all cytokines. IL-35 is a potent immunosuppressive and neuroprotective cytokine, composed of IL-12p35 and EBI3 subunits, and mediates regulatory T-cell functions through its receptor (IL-12Rβ2/gp130) (38). Wang et al. (39) and others have shown that IL-35 promotes the differentiation and expansion of IL-10 producing regulatory T cells, thereby suppressing pro-inflammatory responses (39, 40). IL-35 also prevents blood-brain barrier disruption and suppresses Th17-mediated neuronal damage in experimental autoimmune encephalomyelitis models, establishing CNS-relevant protective mechanisms (41). The marked increase in IL-35 among our BD subjects may reflect an immunoregulatory or compensatory response to systemic inflammation. So, the elevation of IL-35 in BD patients could represent a regulatory, not pro-inflammatory, response. It may affect neuroendocrine signaling or hormones, indirectly influencing bipolar disorder’s complex pathophysiology, aligning with data linking anti-inflammatory cytokines to endocrine and metabolic regulation networks (42, 43). IL-35 also showed small but statistically significant positive associations with CRP and IL-6, indicating that these markers may be modestly upregulated in parallel in response to pro-inflammatory signals. The observed negative relationship between IL-35 and IL-10 represents a weak inverse association and therefore warrants cautious mechanistic consideration. This pattern could be compatible with the presence of partially distinct regulatory pathways or B-cell subsets that predominantly produce IL-35 or IL-10, but this interpretation remains speculative and hypothesis-generating. This aligns with recent studies that identify different B-cell populations, such as the i35-Breg and IL-10-producing B cell (i10Breg) subsets (44, 45). It is worth noting that there was considerable variability in IL-35 levels among BD patients, which could be useful for clustering individuals based on their IL-35 phenotypes. Future studies that further explore the heterogeneity of IL-35-producing regulatory populations are warranted to clarify its contribution into this heterogeneity’s role in BD pathogenesis.

Although IL-39 did not reach statistical significance in primary analyses, the proinflammatory nature of this recently characterized IL-23p19/EBI3 heterodimer warrants mechanistic consideration (20, 46). Unlike IL-27 and IL-35, which exert predominantly immunoregulatory/putatively neuroprotective and immunosuppressive effects (14), IL-39 promotes differentiation and expansion of pathogenic myeloid populations and exhibits pro-inflammatory properties analogous to IL-23 (46). Recent studies by Lu et al. have established that IL-39 is primarily secreted by activated B cells and promotes inflammation by activating STAT3, in contrast to the STAT1-dependent immunoregulatory effects of IL-27 (20). The apparent lack of strong IL-39 dysregulation in our BD sample might be due to the current phase (acute mania) of patients, in which activated B cell responses leading to IL-39 production could differ from those in other phases. Alternatively, IL-39 might act as a marker that becomes dysregulated only in BD groups with pronounced immune disturbance, but this remains speculative and requires confirmation. Studies with larger populations and stratification by mood phase and clinical severity are needed to draw further conclusions about the role of IL-39 in BD.

Unlike the cytokine components of IL-27 and IL-35, EBI3 protein levels did not show similar changes between BD patients and the control group. This finding provides preliminary mechanistic insight, suggesting that although heterodimeric cytokines containing EBI3 appear dysregulated in BD, measuring EBI3 alone may not capture this imbalance accurately. Therefore, EBI3 cannot serve as a surrogate or representative biomarker for all EBI3-containing heterodimeric cytokines. This finding has important implications for prior studies that measured EBI3 polymorphisms or mRNA levels in psychiatric populations (47), as these measures likely do not predict circulating levels of functionally active IL-27 and IL-35 heterodimers. Future studies should employ direct heterodimer measurement via multiplex immunoassays (such as Luminex) rather than component subunit approaches.

A major methodological limitation must be emphasized, BD patients were assessed during moderate-to-severe manic episodes (with mean YMRS scores in the severe range), so we cannot directly compare cytokine patterns across different mood states. As Brietzke et al. study showed (23), that cytokine profiles vary between mania, depression, and euthymia. The notable upward trend in most tested inflammatory mediators among our acute mania sample likely reflects phase-specific rather than trait-like changes. Larger prospective studies examining EBI3-containing cytokines across all affective phases—including well-defined euthymic states and depressive episodes—are needed to distinguish between state- and trait-dependent dysregulation of EBI3 release and to establish clearer links with specific symptoms. Such phase-specific research would greatly enhance understanding of the temporal dynamics of the immune system in BD and help determine whether EBI3-containing cytokines are markers of acute or chronic phases, or both. Additionally, the severity of manic symptoms was not systematically evaluated or controlled, which should be acknowledged as a further limitation since it may have influenced the observed inflammatory patterns.

Paradoxically, increased immunoregulatory/putatively neuroprotective and pro-inflammatory cytokines raise concerns that blunt anti-inflammatory approaches are destined to fail. Instead, targeted immunomodulation of individual dysregulated nodes (e.g., IL-6 antagonist in patients with isolated IL-6 elevation without counterregulatory upregulation of IL-27/IL-35) may optimize therapeutic outcomes. While speculative, IL-27-inducing strategies (e.g., TLR9 agonists or engineered IL-27-producing B cells, currently explored in other neuroimmune contexts) could potentially be of interest for future exploratory research in BD subphenotypes unresponsive to conventional treatments.

## Conclusions

Our results indicate that IL-27 and IL-35 alterations observed in this study are most likely state-related immune changes associated with the acute manic phase of bipolar disorder. These immunoregulatory and putatively neuroprotective cytokines appear to participate in a broader imbalance of immune signaling rather than reflecting a stable trait of the illness. The concurrent elevation of both regulatory and proinflammatory mediators suggests an incomplete compensatory immune response during mania. Given the cross-sectional design and the restriction to acutely manic patients, the present findings should be interpreted with caution and not generalized to other mood states. Larger, longitudinal, and phase-stratified studies are warranted to confirm these observations, assess their temporal dynamics, and clarify whether EBI3-containing cytokines could represent hypothesis-generating candidates for future exploration as state- or trait-related immunomodulatory markers in BD.

## Data Availability

The raw data supporting the conclusions of this article will be made available by the authors, without undue reservation.
